# Ultrasensitive RT-QuIC assay with high sensitivity and specificity for Lewy body-associated synucleinopathies

**DOI:** 10.1007/s00401-020-02160-8

**Published:** 2020-04-27

**Authors:** Marcello Rossi, Niccolò Candelise, Simone Baiardi, Sabina Capellari, Giulia Giannini, Christina D. Orrù, Elena Antelmi, Angela Mammana, Andrew G. Hughson, Giovanna Calandra-Buonaura, Anna Ladogana, Giuseppe Plazzi, Pietro Cortelli, Byron Caughey, Piero Parchi

**Affiliations:** 1 IRCCS, Istituto Delle Scienze Neurologiche di Bologna, Bologna, Italy; 2 Department of Experimental, Diagnostic and Specialty Medicine (DIMES), University of Bologna, Bologna, Italy; 3 Department of Biomedical and Neuromotor Sciences, University of Bologna, Bologna, Italy; 4 LPVD, Rocky Mountain Laboratories, NIAID, NIH, Hamilton, MT USA; 5 Department of Neurosciences, Istituto Superiore di Sanità, Rome, Italy

**Keywords:** α-Synuclein, Parkinson’s disease, Multiple system atrophy, Diagnosis, Prion disease, Biomarker

## Abstract

The clinical diagnosis of synucleinopathies, including Parkinson’s disease (PD), dementia with Lewy bodies (DLB), and multiple system atrophy (MSA), is challenging, especially at an early disease stage, due to the heterogeneous and often non-specific clinical manifestations. The discovery of reliable specific markers for synucleinopathies would consequently be of great aid to the diagnosis and management of these disorders. Real-Time Quaking-Induced Conversion (RT-QuIC) is an ultrasensitive technique that has been previously used to detect self-templating amyloidogenic proteins in the cerebrospinal fluid (CSF) and other biospecimens in prion disease and synucleinopathies. Using a wild-type recombinant α-synuclein as a substrate, we applied RT-QuIC to a large cohort of 439 CSF samples from clinically well-characterized, or post-mortem verified patients with parkinsonism or dementia. Of significance, we also studied patients with isolated REM sleep behavior disorder (iRBD) (*n* = 18) and pure autonomic failure (PAF) (*n* = 28), representing clinical syndromes that are often caused by a synucleinopathy, and may precede the appearance of parkinsonism or cognitive decline. The results show that our RT-QuIC assay can accurately detect α-synuclein seeding activity across the spectrum of Lewy Body (LB)-related disorders (LBD), including DLB, PD, iRBD, and PAF, with an overall sensitivity of 95.3%. In contrast, all but two patients with MSA showed no α-synuclein seeding activity in the applied experimental setting. The analysis of the fluorescence response reflecting the amount of α-synuclein seeds revealed no significant differences between the clinical syndromes associated with LB pathology. Finally, the assay demonstrated 98% specificity in a neuropathological cohort of 101 cases lacking LB pathology. In conclusion, α-synuclein RT-QuIC provides an accurate marker of synucleinopathies linked to LB pathology and may have a pivotal role in the early discrimination and management of affected patients. The finding of no α-synuclein seeding activity in MSA seems to support the current view that MSA and LBD are associated with different conformational strains of α-synuclein.

**Electronic supplementary material:**

The online version of this article (10.1007/s00401-020-02160-8) contains supplementary material, which is available to authorized users.

## Introduction

Synucleinopathies, including Parkinson's disease (PD), dementia with Lewy bodies (DLB), and multiple system atrophy (MSA), are neurodegenerative diseases characterized by the intracellular accumulation of toxic α-synuclein (α-Syn) aggregates [62, 67]. In these pathologies, a conformational change (misfolding) of the α-Syn monomer produces an aggregation nucleus (seed), which can induce the conversion of other α-Syn molecules, forming Lewy bodies (LB), the primary histopathological lesion of PD and DLB, or other oligodendroglial and neuronal cytoplasmic inclusions in MSA [29, 42]. The seeded conversion mechanism, resulting in the self-propagation of misfolded α-Syn, is named nowadays as prion-like, due to the analogy to that initially identified in prion diseases [27, 53, 65].

Clinically, synucleinopathies show a highly variable presentation, response to therapy, and rate of progression. Consequently, the prognosis of these conditions is also very variable. Lewy body disease (LBD), the most common synucleinopathy, comprising DLB and the large majority of PD patients, may manifest clinically with either parkinsonism or cognitive decline (or both), with overlapping symptoms with both atypical parkinsonisms, including progressive supranuclear palsy (PSP), MSA, and corticobasal degeneration (CBD), and neurodegenerative dementias, mainly Alzheimer's disease (AD) [7, 34, 58]. Moreover, the demonstration that clinical conditions such as isolated REM sleep behavior disorder (iRBD) and pure/isolated autonomic failure (PAF) are often caused by a synucleinopathy [16, 33], further complicates the diagnosis of LBD, but, at the same time, provides a potential clinical target for the early recognition of these patients. In particular, the term iRBD was suggested to refer to RBD occurring in the absence of any associated neurological sign or other possible cause [30], a condition characterized by a high phenoconversion rate to an overt synucleinopathy [51]. The identification of a biomarker for synucleinopathies would be, therefore, of great value in clinical practice for the differential and early diagnosis, prognostic assessment, and the monitoring of disease progression. Furthermore, an accurate early diagnosis would optimize the inclusion of patients in clinical trials. Given that imaging with radiotracers, to be applied to research and clinical practice, are available for beta-amyloid and tau proteins but not for α-Syn, the search for reliable markers for synucleinopathies in biological fluids is even more crucial. Unfortunately, misfolded α-Syn, the ideal biomarker candidate, is expressed in biological fluids in concentrations below the detection level of standard analytical techniques [48]. Recently, a novel approach based on the in vitro amplification of the pathogenic protein seed obtained by forcing the conversion of native monomers into their misfolded counterpart provided the basis to overcome this limitation [4, 14, 54, 55]. In one of these seeding amyloid assays (SAAs) called Real-Time Quaking-Induced Conversion (RT-QuIC), the pathogenic seed derived from the CSF, or other biological fluids or tissues, of an affected patient, is incubated with its native counterpart, represented by a recombinant protein [69, 4]. Intermittent shaking is then used to favor the interaction between the seed and the substrate, forcing the conversion of the monomer into its pathogenic counterpart. The course of the reaction is monitored in real time by thioflavin, a fluorescent dye that shows enhanced emission upon binding to cross-β structures, a typical feature of amyloid fibrils. Given its capacity of identifying misfolded forms of prion protein (PrP) from Creutzfeldt–Jakob disease (CJD) CSF with a specificity of 100% and a sensitivity of 95–98%, RT-QuIC has already been included in the diagnostic criteria for the clinical diagnosis of sporadic(s) CJD, the most common human prion disease [70]. By showing that this assay may also accurately discriminate between LBD and other parkinsonisms or dementias unrelated to α-Syn, more recent studies strongly suggested that CSF RT-QuIC can also be applied successfully to synucleinopathies [9, 20, 28, 55, 64], especially to those associated with LB pathology. Here, we expanded these preliminary results by analyzing the largest cohort studied to date, including well-characterized patients with parkinsonism and/or dementia. Moreover, we tested for the first time a significant number of patients affected by iRBD and PAF.

## Materials and methods

The study was conducted according to the revised Declaration of Helsinki and Good Clinical Practice guidelines. Informed consent was given by study participants or the next of kin.

### Patients and controls

We analyzed 439 CSF samples submitted to the Neuropathology Laboratory (NP-Lab) at the Institute of Neurological Sciences of Bologna (ISNB) between 2005 and 2019. The cohort included 122 patients with a post-mortem CNS neuropathological assessment (i.e., “neuropath” cases), and 317 patients in which the clinical diagnosis was reached after a comprehensive evaluation and a significant follow-up (i.e., “clinical” cases). The first group encompassed cases of progressive dementia, more often with a rapidly progressive course, or of atypical parkinsonism, and included the following diagnostic categories: DLB, AD, CJD, Frontotemporal lobar degeneration, MSA, PSP, Encephalitis, Vascular dementia, and other dementias. The “neuropath” group was further divided into LB α-Syn+ and LB α-Syn− subgroups based on protein aggregate assessment by immunohistochemistry (see below for further details).

The “clinical” group included 62 cases lacking symptoms and signs suggesting an underlying progressive neurodegenerative disorder (e.g., chronic headache, narcolepsy type 1 with or without associated RBD; for the complete list see Supplementary Table 1, online resource) and 255 patients fulfilling the current diagnostic criteria of probable or clinically established disease for one of the following disorders/syndromes: iRBD [57], PAF [13, 23], PD [50], MSA [26], PSP [31], CBS [3], DLB [41], and AD/prodromal AD (hence abbreviated as AD) [19]. Moreover, the group included ten patients with the clinical diagnosis of PD (*n* = 8) or DLB (*n* = 2) carried single allele mutations known to be associated with LBD in the glucocerebrosidase gene (*GBA*) (N370S, L444P, R131C, E326K, *n* = 9) or in the Leucine Rich Repeat Kinase 2 gene (*LRRK2*) (G2019S, *n* = 1).

### Clinical assessment and diagnostic criteria

For each patient, we collected the clinical history, and the results of neurological examination(s) and diagnostic investigations, including, when available, brain magnetic resonance imaging (MRI, *n* = 275), cerebral 129I-ioflupane SPECT (DaTSCAN) (*n* = 147), cardiac 123I-metaiodobenzylguanidin (MIBG)-SPECT (*n* = 88), and all-night polysomnography (PSG, *n* = 163) (see also Supplementary Table 2, online resource). For AD and DLB groups, results of neuropsychological examination(s), data of Mini-Mental State Examination (MMSE), and CSF values of AD core biomarkers were also obtained. All patients with suspected autonomic failure (AF) (*n* = 170) were assessed by a battery of cardiovascular reflex tests, including head-up tilt test (10 min at 65°), Valsalva maneuver (40 mm Hg for 15 s), deep breathing (6 breaths/min), and sustained handgrip (one-third of maximal effort for 5 min). All patients with the diagnosis of narcolepsy type 1 (*n* = 15) underwent the multiple sleep latency test, polygraphic assessment of cataplexy, and the evaluation of CSF orexin levels. After CSF collection, most patients belonging to the “clinical” group were longitudinally followed-up at the ISNB [i.e., the follow-up duration was > 2 years in 116 cases (36.6%), and > 1 year in 161 cases (50.8%)]. Only patients with a probable or clinically established (for PD only) diagnosis at last follow-up of iRBD, PAF, PD, MSA, PSP, CBS, DLB, and AD, according to international criteria, were included into the study cohort. The cases fulfilling the criteria for more than one probable disease (e.g., concurrent probable diagnosis of PSP and MSA) were excluded. The term iRBD was used to refer to RBD occurring in the absence of any associated neurological sign or other possible cause [30]. Finally, to increase the accuracy of clinical diagnosis, only patients with PAF presenting with AF as the sole clinical manifestation for at least 5 years were considered [25].

Given that the patients with iRBD and PAF may develop cognitive and/or motor symptoms/signs and convert to other clinical syndromes, including DLB, PD, and MSA, we evaluated separately the phenoconversion process in subjects diagnosed with these prodromic disorders at time of CSF collection but receiving another clinical diagnosis at last follow-up.

### Neuropathological studies

Neuropathological examination was performed using standardized procedures as described [37]. Briefly, according to the autopsy protocol of NP-Lab at ISNB, the brain is divided sagittally and the right hemibrain is fixed in 10% buffered formalin, while the left one is sectioned coronally and then immediately frozen at − 80 °C in sealed plastic bags. The formalin-fixed left hemibrain is serially sectioned in 1 cm slices, and tissue blocks from 24 regions are processed routinely to obtain paraffin-embedded brain tissue blocks [47].

Seven μm-thick sections from each block were stained with hematoxylin–eosin for screening. Also, immunohistochemistry with antibodies specific for α-Syn (LB509, dilution 1:100, Thermo Fisher Scientific, and KM51, dilution 1:500, Novocastra), phospho-tau (p-tau) (AT8, dilution 1:100, Innogenetics), Aβ (4G8, dilution 1:5000, Signet Labs), and PrP (3F4, dilution 1:400, Signet Labs) was applied to all cases using several brain regions, mainly following established consensus criteria [1, 2, 43, 46]. An experienced neuropathologist (P.P.) formulated the final diagnosis, assigned the Braak stage of LB-related pathology [2], and classified each case according to the level of AD neuropathologic change (ABC score) [1, 43].

### CSF collection and analyses

CSF samples were obtained by lumbar puncture (LP) at the L3/L4 or L4/L5 level following a standard procedure. Most CSF samples of the clinical group (299 out of 317) were collected (i.e., LP performed) at the same site (IRCCS-ISNB) and homogeneously handled by trained personnel. In contrast most specimens (116 out of 122) from the patients with post-mortem confirmation derived from general neurologic practice at various sites, since they were recruited through the Italian National Surveillance program for prion disease. All samples, irrespective of their origin, were divided into aliquots of 400–500 μl, and stored in polypropylene tubes at − 80 °C until analysis.

CSF total-tau (t-tau) levels were measured in all cases. For clinical classification purposes, CSF p-tau and Aβ_1–42_ (Aβ42) analyses were limited to AD and DLB cases. Aβ_1–40_ (Aβ40) was evaluated in the AD and control cohorts to calculate the ratio between Aβ42 and Aβ40 according to a previously published formula [(Aβ42)/(Aβ40) × 10] [5]. We measured AD core biomarkers prospectively in a routine clinical setting. CSF t-tau, p-tau, Aβ42, Aβ40, and levels were analyzed using commercially available ELISA kits (INNOTEST htau-Ag, INNOTEST p-tau181, INNOTEST Aβ42, and INNOTEST Aβ40). The results of these analyses are reported in Supplementary Table 3, online resource.

### Purification of human recombinant α-synuclein

Glycerol stock of *E. coli* bacteria containing the vector for wild-type (wt) human α-Syn expression was obtained from Dr. Byron Caughey’s lab. The purification of the recombinant α-Syn was performed as reported [28], with minor modifications. Bacteria from the glycerol stock were inoculated into 5 ml of Luria Broth (LB, Sigma) containing 50 µg/ml of kanamycin (Sigma) and let grow for 4–5 h at 37 °C with continuous agitation at 250 rpm. The initial culture was then added to 1 l of LB containing 50 µg/ml of kanamycin plus the overnight express auto-induction system (Merk-Millipore) in a full baffled flask. Cells were grown in a shaking incubator at 37 °C, 200 rpm overnight. The next day, the culture was split into four 250 ml flasks, and bacteria were harvested by centrifugation at 3200 × *g* for 10 min at 4 °C. The pellet was gently re-suspended in 25 ml osmotic shock buffer containing 40% sucrose, 2 mM EDTA, and 30 mM Tris at pH 7.2 using a 25 ml serological pipette and incubated 10 min at room temperature under mild agitation. Next, the suspension was centrifuged at 7900×*g*, 20 m at 20 °C. The supernatant was discarded, and the pellet was re-suspended in 10 ml of ice-cold water for each pellet. Suspensions were pooled into two 50 ml tubes to a final volume of 20 ml per tube. 20 µl of saturated MgCl_2_ was added to each 20 ml suspension and incubated on ice for 3 min under mild rocking. Next, the suspension was centrifuged at 9000×*g*, 30 min at 4 °C. The pellet was discarded, and the supernatant collected into a 100 ml glass beaker containing a magnetic stir bar. The pH was reduced to pH 3.5 by adding 400–600 µl HCl 1 M and incubated under stirring for 10 min at room temperature. Tubes were centrifuged at 9000 ×*g* for 30 min at 4 °C, the pellet was discarded, and the supernatant collected into a fresh 100 ml glass beaker containing a magnetic stir bar. The pH was adjusted to 7.5 by adding 400–600 µl NaOH 1 M. The protein extract was filtered through a 0.22 µm filter (Merk-Millipore), loaded into a Ni–NTA column (Qiagen) on an NGC chromatographic system (Bio-Rad) and washed with 20 mM Tris, pH 7.5 at room temperature. The column was further washed with 50 mM imidazole in Tris 20 mM, pH 7.5, generating a peak that was not collected. A linear gradient up to 500 mM imidazole in 20 mM Tris, pH 7.5 was performed, and the peak was collected between 30 and 75% of imidazole buffer (150 and 375 mM, respectively). This peak was loaded onto an anion exchange column Q-HP (GE Healthcare) and washed in Tris 20 mM, pH 7.5, followed by another washing in 100 mM NaCl in Tris 20 mM, pH 7.5. A linear gradient up to 500 mM of NaCl in Tris 20 mM pH 7.5 was performed to collect the peak between 300 and 350 mM NaCl. The recovered fractions were pooled together and filtered through a 0.22 µm filter and dialyzed against water overnight at 4 °C using a 3.5 kDa MWCO dialysis membrane (Thermo-Scientific). The next day, the protein was moved into fresh water and dialyzed for four more hours. The protein concentration was measured with a spectrophotometer using a theoretical extinction coefficient at 280 nm of 0.36 (mg/ml)-1/cm. Finally, the protein was lyophilized using a lyophilizer (Thermo-Scientific) for 6 h and stored in aliquots at a final concentration of 1 mg/ml once re-suspended into 550 µl of phosphate buffer (PB) 40 mM, pH 8.0. Lyophilized aliquots were stored at − 80 °C until usage.

### The real-time quaking-induced conversion assay (RT-QuIC)

The RT-QuIC reactions were performed following an established protocol [28]*.* Black 96-well plates with a clear bottom (Nalgene Nunc International) were pre-loaded with six 0.8 mm silica beads (OPS Diagnostics) per well. CSF samples were thawed and vortexed 10 s before use. Fifteen µL of CSF were added as seed to trigger the reaction in 85 µL of buffer containing 40 mM PB, pH 8.0, 170 mM NaCl, 10 mM thioflavin-T (ThT), 0.0015% sodium dodecyl sulfate (SDS), and 0.1 g/l of recombinant α-Syn filtered using a 100 kDa MWCO filter (Pall-Life Sciences). The plate was sealed with a plate sealer film (Nalgene Nunc International) and incubated into Fluostar Omega (BMG Labtech) plate reader at 42 °C with intermittent double orbital shaking at 400 rpm for one minute, followed by 1-min rest. ThT fluorescence measurements were taken every 45 min using 450 nm excitation and 480 nm emission filter. To overcome batch-to-batch variations of α-Syn activity and the intrinsic experimental variability, relative fluorescent units (RFU) for every time point were normalized for the maximum of the intensity of each experimental plate and expressed as a percentage. Samples were run in quadruplicates and deemed positive when at least two out of four replicates reached the threshold, calculated as the average normalized fluorescence value of the first 10 h of the run of the 101 neuropathological control samples, plus 30 standard deviations. The analysis was repeated when only one replicate crossed this threshold.

### Statistical analysis

RT-QuIC relative fluorescence responses were analyzed and plotted using the software Graphpad Prism 8.3. The area under the curve (AUC), the maximum intensity of fluorescence (*I*_max_), and the lag phase were extracted, and the normality of the distribution of these variables from each group was assessed using the Kolmogorov–Smirnov test. The comparison among groups was performed using the one-way ANOVA followed by Tukey’s multiple comparisons post hoc test. *P* values < 0.05 were considered statistically significant. Unless otherwise stated, the error bars indicate the standard deviation (SD).

## Results

### Patients

Demographic data, symptom scores, and numbers and results of performed diagnostic investigations for all patient groups are summarized in Table 1 and Supplementary Tables 2 and 3, online resource.

**Table 1:** Demographic findings of the study cohort

Diagnostic category	*n*	Female (%)	Age at LP (years)	Time between clinical onset and LP (mos)	Follow-up duration (mos)^a^	Time between clinical onset and last visit (years)
Definite NP cohort						
LB-α-Syn+	21	9 (42.9)	75.8 ± 6.3	12.2 ± 28.9	3.9 ± 8.8	2.2 ± 2.5
DLB	14	6 (42.9)	76.9 ± 5.4	17.3 ± 34.1	5.0 ± 10.4	1.7 ± 2.9
Dementia with incidental LB	7	3 (42.9)	74.3 ± 7.7	2.0 ± 1.8	1.1 ± 1.1	0.3 ± 0.2
LB-α-Syn−	101	45 (44.5)	68.5 ± 9.9	3.7 ± 13.4	4.5 ± 12.2	0.7 ± 1.8
AD	17	9 (52.9)	76.4 ± 7.5	1.6 ± 2.5	1.9 ± 2.6	0.3 ± 0.3
PSP	1	0 (0)	64	108	30	11.5
MSA	2	1 (50.0)	62.5 ± 7.8	40.5 ± 54.4	68.5 ± 28.9	9.1 ± 2.1
Syn− controls^b^	81	35 (43.2)	66.9 ± 9.9	1.9 ± 2.1	3.2 ± 7.6	0.4 ± 0.7
Clinical cohort						
DLB	34	9 (26.5)	73.2 ± 7.5	78.8 ± 105.8	14.9 ± 21.4	7.8 ± 8.7
AD	43	22 (52.2)	66.3 ± 7.8	43.4 ± 39.7	12.7 ± 15.8	4.7 ± 3.5
PSP/CBS	30	19 (63.3)	70.7 ± 6.7	47.0 ± 32.9	8.9 ± 15.0	4.7 ± 2.8
MSA	31	12 (38.7)	60.7 ± 8.6	55.2 ± 42.0	23.4 ± 16.3	6.4 ± 3.6
PD^c^	71	19 (26.8)	62.2 ± 8.8	56.8 ± 45.8	31.6 ± 35.9	7.4 ± 4.2
PAF	28	11 (39.3)	65.5 ± 8.5	119.3 ± 62.5	43.9 ± 50.4	13.6 ± 6.9
iRBD	18	6 (33.3)	68.2 ± 7.7	71.5 ± 60.7	10.6 ± 15.1	6.9 ± 4.7
Clinical controls^d^	62	30 (48.4)	53.9 ± 15.4	158.7 ± 152.5	30.2 ± 27.5	15.5 ± 13.5
Total	439	182 (41.5)	65.3 ± 11.4	59.6 ± 85.6	18.3 ± 27.6	6.5 ± 7.5

*LP* lumbar puncture, *DLB* dementia with Lewy bodies, *AD* Alzheimer’s disease, *PD* Parkinson’s disease, *PSP* progressive supranuclear palsy, *CBS* corticobasal syndrome, *MSA* multiple system atrophy, *PAF* pure autonomic failure, *iRBD* isolated REM sleep behaviour disorder, *α-Syn* α-synuclein

^a^The follow-up duration was calculated from LP to the last visit (or death)

^b^Neuropathological cases diagnosed as Creutzfeldt–Jakob disease (*n* = 51), malignancy (*n* = 3), vascular disease (*n* = 6), encephalitis (*n* = 10), Wernicke’s encephalopathy (*n* = 3), frontotemporal lobar degeneration plus amyotrophic lateral sclerosis (*n* = 1), non-specified tauopathy (*n* = 1), and dementia lacking distinctive pathology (*n* = 6)

^c^53 clinically established and 18 probable cases

^d^For a detailed list, see Supplementary Table 1 (online resource)

### CSF Syn RT-QuIC identifies Lewy body-related pathology with high sensitivity and specificity

**Fig. 1:**
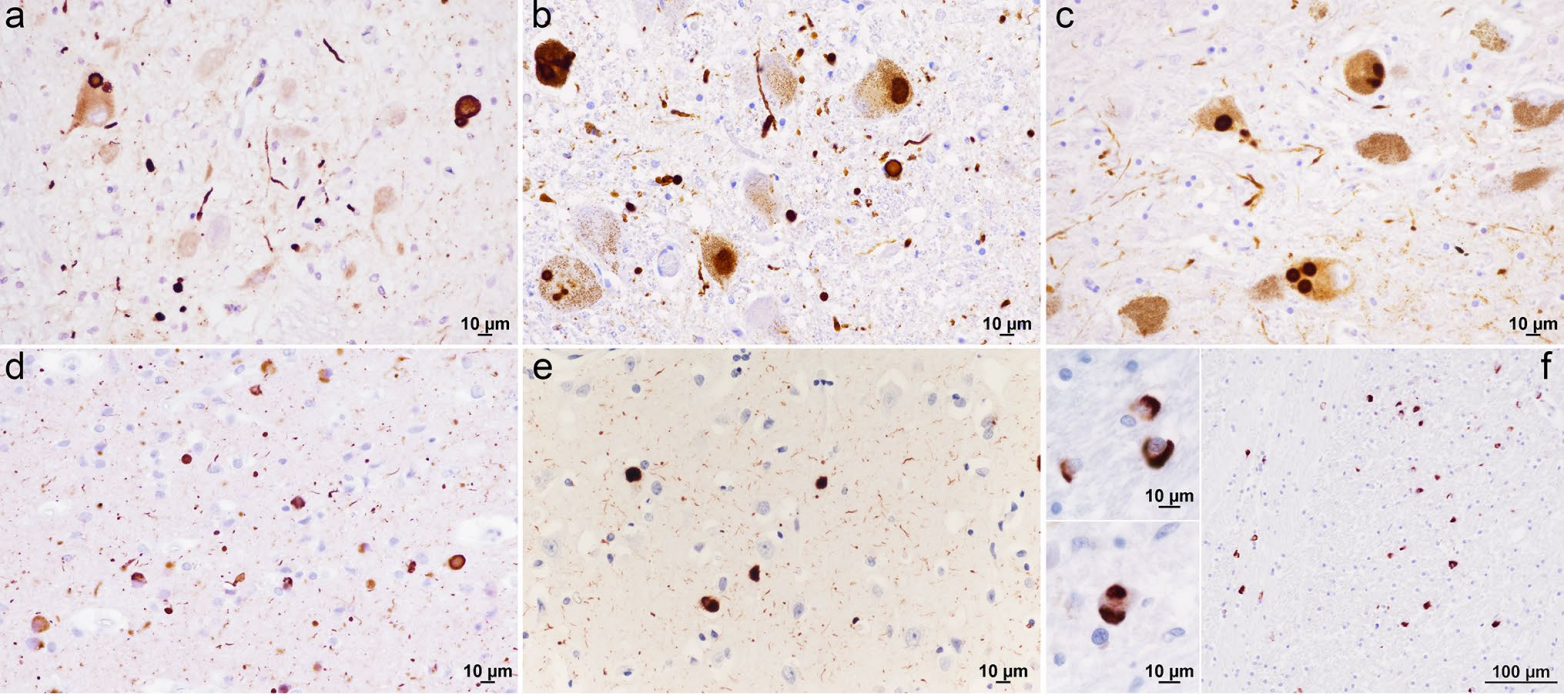
Distinctive α-Syn pathology in patients with LBD and MSA. **a** Moderate number of LBs and Lewy neurites in the medulla of a case with incidental LB pathology (case #18); **b** numerous pathological aggregates of α-Syn in the locus coeruleus (case #8); **c** single and multiple LB inclusions in the neurons of substantia nigra (case #8); **d** numerous LBs and positive dendrites in the amygdala (case #3); **e** moderate number of LBs and positive dendrites in the temporal neocortex (case #1); **f** numerous glial cytoplasmic inclusions (GCIs) in oligodendroglia in the internal capsule of a patient with MSA-P; the two boxes on the left show high magnifications of two GCIs (basal ganglia, upper box) and one neuronal cytoplasmic inclusion (pons, lower box)

**Fig. 2:**
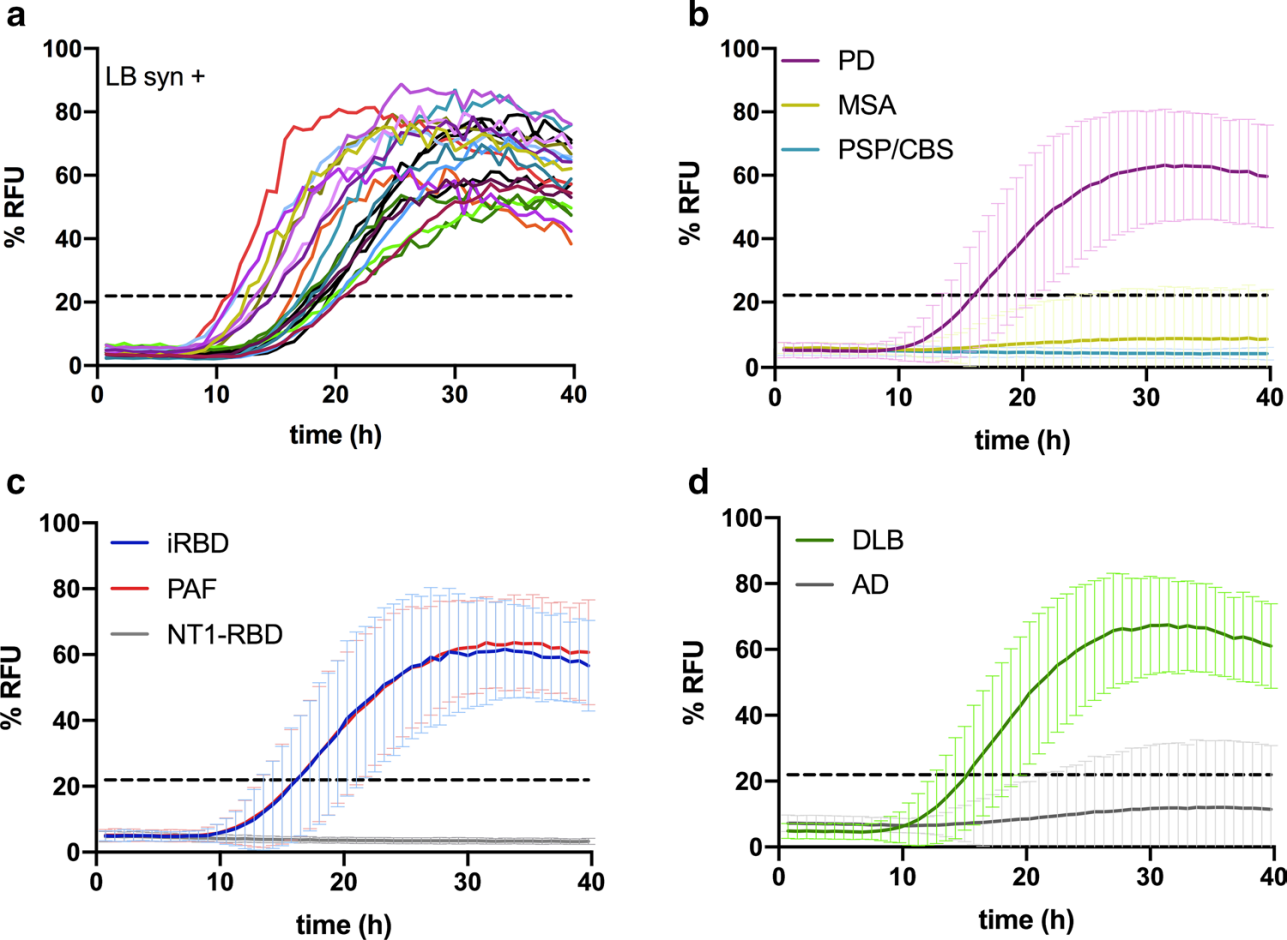
Kinetic curves of α-Syn seeding activity measured by RT-QuIC. **a** Seeding activity of neuropathologically confirmed LB α-Syn + cases (*n* = 21). Each curve depicts the average of quadruplicates. Standard deviation (SD) was hidden to make the image more readable; **b** comparison among PD (purple line, *n* = 71), MSA (yellow line, *n* = 33), and PSP/CBS (pale blue line, *n* = 31) clinical cases; **c** comparison among iRBD (blue line, *n* = 18), PAF (red line, *n* = 28), and narcolepsy type 1 plus RBD (gray line, *n* = 11) clinical cases; **d** comparison between DLB (green line, *n* = 48) and AD (dark gray line, *n* = 60). Clinical and neuropathologically confirmed cases are grouped together. Each curve represents the average of the group, error bars indicate the SD, and the black dashed line indicates the threshold. RFU values are normalized to percentage against the maximum intensity of fluorescence of the respective experimental plate

Our preliminary analyses using the wild-type α-Syn and the protocol published by Groveman et al. [28] showed high sensitivity of the RT-QuIC assay in patients with LBD. Thus, to thoroughly and reliably assess the accuracy of the test, we initially examined CSF samples from 21 subjects demonstrating various extents of LB-related pathology at post-mortem examination (i.e., Braak stage 1–6, Fig. 1, and Table 2), 101 subjects lacking LB-related pathology assessed by immunostaining, and 62 subjects who did not manifest symptoms or signs suggestive of a progressive neurological disorder. In the neuropathologically verified cohort (Fig. 2a), the CSF α-Syn RT-QuIC yielded an overall specificity of 98.0% and a sensitivity of 95.2% (100% in definite DLB), whereas the specificity calculated on the clinical controls was 98.4% (61/62 negatives), or 97.6% (40/41 negatives) when only the subgroup of controls age-matched (64.0 ± 6.2 years) with the iRBD, PAF, and PD groups was considered. Unexpected positive results were limited to two cases with a primary neuropathological diagnosis of Wernicke’s encephalopathy and Alzheimer’s disease, which showed no detectable α-Syn deposits, despite the positivity by RT-QuIC, and one 76 years old clinical control with a diagnosis of peripheral sensory neuropathy.

**Table 2:** Demographic and neuropathological features of definite cases with LB-Syn+

List of cases	Sex	Age at LP	Age at death	Primary NP diagnosis	Secondary NP diagnosis	McKeith stage	Braak stage
Case #1	F	79	79	DLB	AD (low)	Neocortical	6
Case #2	F	81	81	DLB	AD (low)	Neocortical	6
Case #3	F	79	81	DLB	AD (intermediate)	Neocortical	6
Case #4	M	77	77	DLB	AD (intermediate)	Neocortical	6
Case #5	M	63	65	DLB	AD (high)	Neocortical	6
Case #6	M	77	77	DLB	AD (intermediate)	Neocortical	6
Case #7	F	81	81	DLB	AD (intermediate)	Neocortical	6
Case #8	M	80	80	DLB	AD (intermediate)	Neocortical	6
Case #9	F	82	83	DLB	AD (intermediate)	Neocortical	5
Case #10	M	71	72	DLB	AD (low)	Neocortical	5
Case #11	M	73	73	DLB	AD (low)	Neocortical	5
Case #12	F	76	77	DLB	AD (intermediate), CAA, subcortical arteriosclerotic encephalopathy	Limbic	4
Case #13	M	73	73	DLB	AD (intermediate), CAA	Limbic	4
Case #14	M	83	83	DLB	AD (intermediate), CAA	Limbic	4
Case #15^a^	M	67	67	sCJD MM1^b^	DLB	Neocortical	5
Case #16^a^	F	73	73	sCJD VV2^b^	DLB, AD (intermediate), CAA	Neocortical	5
Case #17^a^	F	75	76	Primary CNS lymphoma	PART^c^	Limbic	4
Case #18^a^	M	87	87	Metabolic encephalopathy	AD (low), lacunar stroke	Brainstem	3
Case #19^a^	M	72	73	sCJD VV2^b^	AD (low)	Brainstem	2
Case #20^a^	M	81	81	sCJD MV2K + 2C^b^	AD (low)	Brainstem	1
Case #21^a^	F	65	65	sCJD MM1^b^	None	Brainstem	1

*LP* lumbar puncture, *NP* neuropathologic, *DLB* dementia with Lewy bodies, *AD* Alzheimer’s disease, *CAA* cerebral amyloid angiopathy, *CNS* central nervous system, *sCJD* sporadic Creutzfeldt–Jakob disease, *PART* primary age-related tauopathy

^a^Dementia with incidental LB co-pathology

^b^sCJD subtypes were defined in accordance with Parchi et al. [47]

^c^PART was diagnosed according to Crary et al. [18]

Having established the very high specificity and sensitivity of the assay as a marker of LB-related pathology in a neuropathologically confirmed cohort and in non-neurodegenerative clinical controls, we then examined the performance of the assay in a well-characterized patient population representing the whole clinical spectrum of LBD, namely parkinsonism, dementia, iRBD, and PAF.

### Performance of α-Syn RT-QuIC in patients with parkinsonism

To evaluate the performance of the RT-QuIC in patients with parkinsonism, we compared well-characterized patients, fulfilling clinical criteria for a probable or clinically established disease of, respectively, PD, MSA, and PSP/CBS. Positive seeding activity was consistently detected only in PD, but neither in PSP/CBS nor, unexpectedly, in MSA (Fig. 2b). In the probable PD cohort, the assay yielded a sensitivity of 94.4%, whereas the specificity was 100% against patients with PSP/CBS and 93.5% against those with MSA (Table 3). Among the 67 PD patients who tested positive by RT-QuIC, 48 (71.6%) showed a full 4/4 positive response, 13 (19.4%) gave 3/4, and 6 (8.9%) gave 2/4 (Supplementary Table 4, online resource). All the patients carrying a mutation in *GBA* tested positive by RT-QuIC, whereas the one carrying the mutation in *LRRK2* did not show any α-Syn aggregation. The latter result is in line with those of a previous study demonstrating a much lower sensitivity of the RT-QuIC in *LRRK2*-PD than in idiopathic PD [24]. Discordant results were limited to two patients with probable MSA who showed α-Syn seeding activity and three with probable idiopathic PD who tested negative. A detailed analysis of the clinical features of patients yielding unexpected results revealed that in the three idiopathic PD subjects, the disease severity was mild (baseline score at the UPDRS scale section III: 16, 14, and 8), and two of them presented with isolated, unilateral tremor. One patient with the diagnosis of probable MSA had an “atypical” long clinical history of more than 10 years, characterized by a slowly progressive cerebellar syndrome and late appearance of parkinsonism and AF. In contrast, the other one had typical MSA features, including the early association of autonomic, pyramidal, extrapyramidal and cerebellar signs, and a more rapid progression of symptoms.

**Table 3:** Sensitivity and specificity of the α-Syn RT-QuIC assay across the diagnostic groups

Diagnostic category	*n*	Pos	Neg	Sensitivity (%)	Specificity (%)
Definite NP cohort					
LB-α-Syn+	21	20	1	**95.2**	
Definite DLB	14	14	0	100	
Dementia with incidental LB	7	6	1	85.7	
LB-α-Syn−	101	2	99		**98.0**
AD	17	1	16		94.1
PSP	1	0	1		100
MSA	2	0	2		100
Syn− controls	81	1	80		98.8
Clinical cohort					
AD	43	7	36		83.7
Clinical controls	62	1	61		98.4
PSP/CBS	30	0	30		100
MSA	31	2	29		93.5
DLB	34	33	1	97.1	
PD	71	67	4	94.4	
iRBD	18	18	0	100	
PAF	28	26	2	92.9	
All LB-related synucleinopathies^a^	172	164	8	**95.3**	

*Pos* positive, *Neg* negative, *AD* Alzheimer’s disease, *PSP* progressive supranuclear palsy, *CBS* corticobasal syndrome, *MSA* multiple system atrophy, *DLB* Dementia with Lewy bodies, *PD* Parkinson’s disease, *iRBD* isolated REM sleep behaviour disorder, *PAF* pure autonomic failure, *α-Syn* α-synuclein

Bold symbol highlights the sensitivity of the assay in the two most significant diagnostic group and its specificity against the LB-associated α-Syn negative controls

^a^Both neuropathologically confirmed and clinical cases

Overall, these results indicate that our RT-QuIC set-up can accurately detect α-Syn seeding activity in the CSF of subjects with probable/clinically established PD and can discriminate with high precision typical PD from atypical parkinsonism such as PSP/CBS, and MSA.

### Performance of α-Syn RT-QuIC in patients with prodromal syndromes: iRBD and PAF

Given the lack of clinical criteria that reliably establish the “probable” LB-related etiology of these syndromes at disease onset, we focused the analysis on clinically well-characterized patients (see “Materials and methods”) who received an adequate clinical follow-up.

In the iRBD cohort, the assay showed α-Syn seeding activity in 18/18 patients (Fig. 2c, blue curve), thus yielding a sensitivity of 100%. Among these patients, 11 (61.1%) showed a full 4/4 positive response, 5 (27.8%) gave 3/4, and 2 (11.1%) gave 2/4 (Supplementary Table 4, online resource). Interestingly, one patient with the diagnosis of probable MSA at follow-up who had only RBD and AF at the time of LP tested negative by RT-QuIC. Furthermore, the assay did not show any seeding activity in 11/11 narcoleptic patients affected by RBD features, resulting in a specificity of 100% towards this clinical mimic.

Similarly, in the PAF cohort (Fig. 2c, red curve), 26 out of 28 samples showed α-Syn seeding activity, resulting in a sensitivity of 92.9%. Among the 26 positive patients, 22 (84.6%) showed a full 4/4 positive response, 3 (11.5%) gave 3/4, and only 1 (3.8%) gave 2/4 (Supplementary Table 4, online resource). Of notice, of the two PAF subjects tested negative by RT-QuIC, one had clinical history relevant for intermittent diplopia and positivity of antiganglioside GQ1b antibody in serum indirectly suggesting a possible underlying autoimmune etiology. At the same time, the other one did not show any neurologic symptoms, but demonstrated a normal adrenergic cardiac innervation at MIBG-SPECT, a finding that has been consistently associated with MSA, but not with PD and DLB [11, 63].

Interestingly, two patients of the probable MSA group, who had a diagnosis of PAF or PAF plus RBD at the time of LP, did not show any α-Syn seeding activity. More details about the patients who underwent phenoconversion from prodromic syndromes to MSA, PD, and DLB are shown in Supplementary Table 5, online resource.

Overall, these results indicate that RT-QuIC can reliably detect pathogenic species of α-Syn in the CSF of patients affected by PAF and iRBD, and can discriminate them from RBD associated with narcolepsy or MSA.

### Performance of wt-Syn RT-QuIC in patients with dementia

Finally, we assessed the ability of the RT-QuIC to detect α-Syn seeding activity in cases with dementia. To this aim, we grouped the certain cases who received post-mortem examination, with the clinical cohorts of probable AD and probable DLB. In this population (Fig. 1d, Table 3), the assay yielded a sensitivity of 97.9% for DLB (100% in the definite group, and 97.1% in the clinical group), whereas 8 out of 60 AD cases tested gave a positive signal, resulting in 86.7% specificity towards AD (83.7% when only the clinical AD cases were considered). Among patients with definite or probable DLB, who tested positive by RT-QuIC, 39 (83.0%) showed a full 4/4 positive response, 6 (12.7%) gave 3/4, and only 2 (4.2%) gave 2/4 (Supplementary Table 4, online resource).

All combined, these results indicate that the α-Syn RT-QuIC is a highly sensitive and specific assay for the detection of α-Syn seeding activity in the CSF of subjects with either probable or confirmed LB-related pathology. As summarized in Table 3, the overall sensitivity of the assay in identifying definite (*n* = 20) or probable (*n* = 144) LB-related synucleinopathies (DLB + PD + iRBD + PAF) was 95.3% (164/172).

### Comparison of relative RT-QuIC seeding activity across the LBD clinical spectrum

**Fig. 3:**
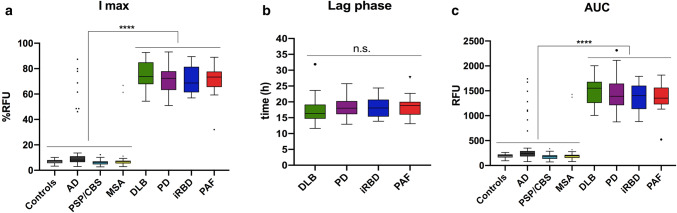
Parameters describing the kinetics of α-Syn aggregation in the RT-QuIC assay. **a** Comparison among the *I*_max_ of each group; **b** comparison of the lag phase of the positive groups; **c** comparison of the AUC of each diagnostic group analyzed. Box plot colors match those represented in the kinetic curves. Box plots show the range and the average of the distribution. Symbols indicate the outlier according to Tukey test. Statistical analyses were conducted using one-way ANOVA, resulting in a significance of *p* < 0.0001 (****) between each of the positive groups against the negatives

We also analyzed the main parameters describing the kinetic curve of the RT-QuIC. As expected, there was a significant difference in both the *I*_max_ (Fig. 3a) and the AUC (Fig. 3c) between each of the LB-related groups (PD, DLB, iRBD, and PAF) and the “negative” groups (MSA, PSP/CBS, AD, and clinical controls). However, within LB positive groups, we found no statistically significant differences in any of the parameters mentioned above, although the DLB group showed a slight trend towards a higher *I*_max_ and AUC. Overall, the mean maximum fluorescence intensity of the “positive” groups was approximately 80% (relative to the *I*_max_ reached in every single experiment), whereas the lag phase (Fig. 3b) varied between 10 and 24 h, regardless the pathological group.

These results suggest that, although the α-Syn RT-QuIC can readily set apart LB-related pathologies from LB independent pathologies, it has no discriminatory power among synucleinopathies, with the relevant exception of the MSA cases, which behaved as the negative control group in this experimental setting.

### Analysis of the assay reproducibility

We ran positive and negative controls on each plate. After normalization, there were no significant differences between intra-batch and batch-to-batch runs in the main parameters describing the kinetic curve of the RT-QuIC (i.e., *I*_max_, lag phase, and AUC), using the same positive or negative control sample (Supplementary Fig., online resource). The intra-batch coefficient of variation (CV) of the *I*_max_, the most significant parameter to determine whether a sample is “positive” or “negative” varied between 7.4 and 22.2% (median = 13.7%) in the positive control and between 7.8 and 39.6% (median = 11.9%) in the negative control, while the inter-batch CVs for the same parameter was, respectively, 17.6% in the positive control, and 36.4% in the negative one. All CVs for the other two parameters (lag phase and AUC) are provided in supplementary Table 8 (online resource).

In a total of 439 sample runs, an unclear result (one positive well) occurred 28 times (6.4%), and in most cases (85.7%) involved samples yielding a negative result at the second test (Supplementary Table 6, online resource). Finally, all but three of the 22 positive samples that we tested three times using different substrate batches, confirmed the positive result of the first run in both repetitions (Supplementary Table 7, online resource). As the only exceptions, two samples with a two out of four positivity gave unclear results in the second run, but confirmed the positivity in the third one, while a third sample gave a one out of two as the third result after two positive results. However, in none of them, there was a change in the diagnostic decision (positive vs. negative).

## Discussion

Emerging ultrasensitive techniques investigating the seeding aggregation of various amyloidogenic proteins currently provide the highest expectations towards the goal of improving accuracy and early diagnosis of neurodegenerative diseases, especially of those lacking reliable diagnostic biomarkers such as DLB, PD, and the Frontotemporal lobar degeneration spectrum. Following the success obtained in prion disease [4, 17, 22, 40, 45, 69], these assays have recently shown promising pilot results also for synucleinopathies [20, 28]. In these studies, α-Syn RT-QuIC has been applied to CSF samples from PD, DLB, and MSA patients using different experimental set-ups [20, 28]. Furthermore, protein misfolding cyclic amplification, another SAA, has also been exploited to demonstrate α-Syn aggregates in the CSF of PD patients [15, 60]. While the first two studies with RT-QuIC demonstrated a full specificity of the assay, subsequent studies reported a significant decrease in specificity ranging from 82 to 92% [24, 64]. Thus, the impact on diagnostic criteria and their clinical applicability is still hampered by the limited number of patients analyzed, especially in the early disease stages, and the less than optimal overall diagnostic accuracy obtained in some replication studies [9, 24, 64]. In the present study, we obtained an almost full specificity in the largest cohort studied to date, including many cases verified neuropathologically post-mortem and, for the first time, a significant number of patients affected by prodromal syndromes such as iRBD and PAF. Overall, our results demonstrate that, in the applied experimental conditions, the positivity of CSF α-Syn RT-QuIC represents a very robust biomarker for LB-related disorders across their entire clinical spectrum. We believe that the accuracy of our results depends on the experimental set-up, and, most critically, on the chosen recombinant substrate. Indeed, at variance with all the RT-QuIC studies with suboptimal specificity cited above, our assay demonstrated a reduced lag phase, which, according to the previous studies in CJD, significantly increases the performance of the assay [22, 45]. Moreover, this is the second study using the experimental conditions put forward by Groveman et al. [28], which also demonstrated a very high specificity. To obtain an accurate estimate of the diagnostic value of our assay, we selected, in addition to neuropathologically verified cases, well-characterized clinical PD and DLB cohorts with a significant clinical follow-up. However, given the much higher complexity of the real clinical world, it is foreseeable that RT-QuIC will also detect α-Syn seeding activity in a significant percentage of patients not fulfilling current criteria for probable PD or DLB, resulting in a significant impact on current diagnostic criteria for both disorders. Similarly, RT-QuIC will likely play a significant role in the identification of patients with prodromal PD and DLB, which is of high interest for clinical studies with disease-modifying drugs. Indeed, it is well established that by the time patients receive a diagnosis, they are typically far along in their disease progression, and that the early diagnosis will be essential for maximizing the chances of effective disease-modifying treatment. It is also increasingly clear that PAF and iRBD are the most useful clinical syndromes for identifying prodromal synucleinopathies. In one 4-year prospective cohort, individuals who presented initially with PAF had a 34% risk for converting to LBD or MSA, especially if they also had RBD [35]. Similarly, patients with iRBD who are followed-up long enough, almost invariably go on to develop PD or other synucleinopathies [32, 51]. We have shown that the CSF of patients with PAF and iRBD harbors α-Syn seeding activity that is comparable to those with PD or DLB. This significant result confirms that most patients with iRBD and PAF have an LBD, but, most importantly, demonstrates that patients with LB-related synucleinopathies can be identified early in the course of the disease with high accuracy.

Although the positive versus negative results represent the most informative RT-QuIC readout, the assay may also provide “quantitative” data based on the analysis of the average kinetics of seeding. To this aim, we measured the lag phase, the fluorescence peak, and the area under the curve of the fluorescence response. Differences in the average values of these parameters between CSFs from patients with various clinical syndromes may be potentially useful for diagnostics and even indicative of a “strain” difference, as previously observed with different types of CJD in prion RT-QuIC reactions [6, 21, 39, 44]. Indeed, the existence of α-Syn strains, which may account for or contribute to the phenotypic heterogeneity of synucleinopathies, has been proposed both in vitro [10, 12, 38] and in vivo [49, 52].

Although a limited difference, though statistically significant, in seeding activity between PD and DLB was previously noted in a relatively small patient cohort [28], we could not confirm this finding in our larger cohort. Indeed, both the lag phase and the overall fluorescence curve response did not vary significantly between different LB-related clinical syndromes. Thus, at least in the current setting, α-Syn RT-QuIC neither distinguished between LBD clinical phenotypes nor provided insight into the progression of symptomatic patients within the LBD clinical spectrum. Accordingly, α-Syn seeding activity in the CSF does not appear significantly correlated with the extent of LB-related pathology within the CNS once the LB pathology has spread sufficiently to cause a neurological syndrome. However, additional experiments, including end-point dilution sample analyses, will be needed to establish more accurately the relative amount of seeding activity in each patient group [56]. Our preliminary data obtained in a few cases with incidental focal LB pathology limited to the medulla indicated that α-Syn seeding activity in the CSF is already detectable at this stage, although possibly with reduced sensitivity.

A significant strength of this study is the use of a large cohort pathologically verified cases to demonstrate the high specificity of the assay for LB-related pathology. Indeed, we only had two discordant results. No α-Syn deposits were detected by immunostaining in these cases; however, this analysis did not include the spinal cord, which can be an initial site of α-Syn accumulation. Thus, we cannot completely rule out the presence of α-Syn pathology in these two patients. The observed 100% specificity of the assay in the pre-senile control group of subjects lacking progressive neurological signs seems to exclude spontaneous aggregation as the leading cause of false-positive results, thereby supporting the possibility that at least some of the few discordant results which we obtained may represent real evidence of positive α-Syn seeding activity. Indeed, given the relative frequency of synucleinopathies in the elderly population, even in the absence of neurological symptoms or signs [8, 36, 66], the occurrence of LBs as co-pathology should also be considered. Thus, a few AD cases that were positive by RT-QuIC might also harbor LBs, at least as significant co-pathology.

On the opposite side, the relatively low number of neuropathologically verified LBD cases, and, possibly, the lack of a group of fully healthy controls may be considered the most significant limitation of our study. However, the selection of a large clinical cohort of well-characterized patients with a significant clinical follow-up has likely reduced the discrepancy between clinical and post-mortem diagnosis at a minimum.

The finding that the vast majority of MSA patients, a bona fide synucleinopathy, did not show α-Syn seeding activity deserves a further comment. In the only previous study of this kind [64], the α-Syn RT-QuIC sensitivity for MSA was also quite low (35.2%, 6/17 positive cases), although not as low as in our experience. In another study, α-Syn protein misfolding cyclic amplification (PMCA) discriminated both PD (88.5%) and MSA (80%, 8/10 positive cases) patients from controls and other neurodegenerative disorders. However, the mean maximum fluorescence signal from MSA samples was much smaller than the corresponding signal from patients with PD [60]. Along this line, the same investigators recently exploited the PMCA methodology to demonstrate that the different reactivity of α-Syn between PD and MSA can be used to discriminate between samples of CSF from the two groups of patients with an accuracy of 95% [61]. The most likely explanation of our and previous results is that the α-Syn pathological conformer associated with MSA has structural constraints that limited its template activity in the tested RT-QuIC, the extent of which may vary according to the specific experimental conditions. Significant structural differences in the α-Syn aggregates between MSA and PD are, indeed, increasingly recognized [59, 61, 68]. In this scenario, our data would strongly support the hypothesis that MSA and LBD are linked to two distinct “strains” of α-Syn aggregates, while on the opposite, they would suggest, but not prove, that a similar α-Syn aggregate conformation characterizes the whole spectrum of LBD.

As an additional implication for the pathobiology of α-synucleinopathies, the demonstration that the CSF of patients belonging to the whole spectrum of LBD is capable of seeding the amyloid aggregation of wild-type α-Syn with high efficiency strongly supports the prion-like nature of these disorders.

In summary, here, we have shown the α-Syn RT-QuIC provides a very robust biomarker for LB-related disorders, a finding that supports its rapid implementation in clinical practice, especially in research centers participating in patient selection for clinical trials. Given the high accuracy with which the assay identifies the LB-associated α-Syn pathology, it might also be the time to update the current nomenclature for iRBD, PAF, and PD, to introduce the eponym LB to each of these syndromes (i.e., PAF-LB, iRBD-LB, and PD-LB), to correctly classify patients with in vivo evidence for LB-related pathology obtained employing RT-QuIC or other SAAs. In this context, the application of RT-QuIC to genetically determined PD cases will also significantly contribute to establishing which are the genetic defects causing an LB-related PD from those causing the degeneration in the substantia nigra without LB formation.

Future studies should establish the impact of α-Syn RT-QuIC on current diagnostic criteria across the spectrum of LB-related disorders, determine the performance of other diagnostic specimens such as skin biopsies and olfactory brushings in comparison with CSF, evaluate the correlation between levels of α-Syn seeding activity and disease progression, and search for variations in the RT-QuIC set-up that might specifically detect α-Syn seeding activity in MSA.

## Electronic supplementary material

Below is the link to the electronic supplementary material.

Supplementary file1 (PDF 433 kb)
