# Correction to: Ultrasensitive RT-QuIC assay with high sensitivity and specificity for Lewy body-associated synucleinopathies

**DOI:** 10.1007/s00401-020-02170-6

**Published:** 2020-06-17

**Authors:** Marcello Rossi, Niccolò Candelise, Simone Baiardi, Sabina Capellari, Giulia Giannini, Christina D. Orrù, Elena Antelmi, Angela Mammana, Andrew G. Hughson, Giovanna Calandra-Buonaura, Anna Ladogana, Giuseppe Plazzi, Pietro Cortelli, Byron Caughey, Piero Parchi

**Affiliations:** 1 IRCCS, Istituto Delle Scienze Neurologiche di Bologna, Bologna, Italy; 2 Department of Experimental, Diagnostic and Specialty Medicine (DIMES), University of Bologna, Bologna, Italy; 3 Department of Biomedical and Neuromotor Sciences, University of Bologna, Bologna, Italy; 4 LPVD, Rocky Mountain Laboratories, NIAID, NIH, Hamilton, MT USA; 5 Department of Neurosciences, Istituto Superiore di Sanità, Rome, Italy

## Correction to: Acta Neuropathologica https://doi.org/10.1007/s00401-020-02160-8

The article Ultrasensitive RT‑QuIC assay with high sensitivity and specificity for Lewy body‑associated synucleinopathies, written by Marcello Rossi, Niccolò Candelise, Simone Baiardi, Sabina Capellari, Giulia Giannini, Christina D. Orrù, Elena Antelmi, Angela Mammana, Andrew G. Hughson, Giovanna Calandra‑Buonaura, Anna Ladogana, Giuseppe Plazzi, Pietro Cortelli, Byron Caughey and Piero Parchi, was originally published electronically on the publisher’s internet portal on 27 April 2020 without open access.With the author(s)’ decision to opt for Open Choice the copyright of the article changed on 26 May 2020 to © The Author(s) 2020 and the article is forthwith distributed under a Creative Commons Attribution 4.0 International License (https://creativecommons.org/licenses/by/4.0/), which permits use, sharing, adaptation, distribution and reproduction in any medium or format, as long as you give appropriate credit to the original author(s) and the source, provide a link to the Creative Commons licence, and indicate if changes were made.

The original article has been corrected.

