# Supplementary material for: Ultrasensitive RT-QuIC assay with high sensitivity and specificity for Lewy body-associated synucleinopathies
Source: Acta Neuropathol. 2020 Apr 27;140(1):49–62. doi: 10.1007/s00401-020-02160-8 (PMC7299922; doi:10.1007/s00401-020-02160-8)
Supplement: Supplementary file 1 — Supplementary file1 (PDF 433 kb) [file 401_2020_2160_MOESM1_ESM.pdf]

Ultrasensitive RT-QuIC assay with high sensitivity and specificity for Lewy Body associated synucleinopathies

Marcello Rossi, Niccolò Candelise, Simone Baiardi, Sabina Capellari, Giulia Giannini, Christina D. Orrù, Elena Antelmi, Angela Mammana, Andrew G. Hughson, Giovanna Calandra-Buonaura, Anna Ladogana, Giuseppe Plazzi, Pietro Cortelli, Byron Caughey, Piero Parchi.

Supplementary materials.

Supplementary Table 1. Diagnostic categories in the clinical control group.

**Diagnostic categories**	**n**	**%**
Chronic headache	29	46.8
Narcolepsy type 1 plus REM sleep behavior disorder	11	17.7
Narcolepsy type 1	4	6.4
Peripheral polyneuropathy	3	4.8
Periodic limb movement disorder	3	4.8
Delirium due to drug/alcohol abuse	2	3.2
Myelopathy due to vitamin B12 deficiency	2	3.2
Benign focal amyotrophy	1	1.6
Optic atrophy	1	1.6
Epilepsy	1	1.6
Subjective cognitive impairment	1	1.6
Psychiatric disorder	1	1.6
CNS neoplasia (meningioma)	1	1.6
Poliomyelitis	1	1.6
Stiff limb syndrome	1	1.6
**Total**	62	100

Supplementary Table 2. Overview of MMSE results and diagnostic investigations performed in the clinical cohort.

	DLB (n=34)	PD (n=71)	AD (n=43)	PSP/CBS (n=30)	MSA (n=31)	PAF (n=28)	iRBD (n=18)	Clinical ctrl (n=62)
**Brain MRI (%)**	25 (73.5)	61 (85.9)	36 (83.7)	27 (90.0)	31 (100)	26 (92.8)	16 (88.9)	53 (85.5)
**SPECT DaTSCAN (%)**	19 (55.9)	55 (77.5)	-	15 (50.0)	24 (77.4)	16 (57.1)	14 (77.8)	4 (6.4)
**Cardiac ^{123}I-MIBG scintigraphy (%)**	15 (44.1)	30 (42.2)	-	5 (16.7)	19 (61.3)	19 (67.8)	-	-
**Polysomnography (%)**	17 (50.0)	47 (66.2)	1 (2.3)	9 (30.0)	29 (93.5)	22 (78.6)	18 (100)	20 (32.2)
**Autonomic tests (%)**	24 (70.6)	60 (84.5)	-	17 (56.7)	30 (96.8)	28 (100)	11 (61.1)	-
**Neuropsychological assessment (%)**	32 (94.1)	61 (85.9)	43 (100)	27 (90.0)	25 (80.6)	14 (50.0)	18 (100)	7 (11.3)
**MMSE (yrs from disease onset)**	21.6±5.6 (7.9±9.9)	27.7±3.8 (5.0±4.0)	21.9±5.4 (3.8±2.9)	27.1±2.5 (3.9±2.6)	28.4±1.8 (4.5±3.4)	28.1±1.9 (12.0±5.6)	29.1±1.3 (6.2±5.1)	29.2±0.8 (3.7±4.7)

List of abbreviations: DLB, dementia with Lewy bodies; PD, Parkinson's disease; AD, Alzheimer's disease; PSP, progressive supranuclear palsy; CBS, corticobasal syndrome; PAF, pure autonomic failure; iRBD, isolated REM sleep behaviour disorder; ctrl, controls; MRI, magnetic resonance imaging; MMSE, Mini-Mental State Examination.

Supplementary Table 3. Results of CSF biomarkers (A/T/N system) across the diagnostic groups.

	t-tau	p-tau	Aβ42	Aβ40	Aβ42/Aβ40x10
**AD**	773.3±508.5 (55)	91.7±37.9 (53)	450.0±207.4 (53)	8820.3±4081.8 (47)	0.51±.0.17 (47)
**DLB**	445.6±821.9 (45)	48.1±25.1 (33)	570.8±385.4 (34)	6181.8±3339.9 (26)	0.82±0.37 (26)
**PSP/CBS**	203.6±84.7 (31)	33.3±11.5 (19)	806.8±344.5 (21)	5755.8±2350.3 (9)	1.12±0.26 (9)
**PAF**	220.6±145.9 (22)	35.2±9.0 (10)	1034.4±558.9 (11)	7572.2±3262.3 (9)	1.43±0.67 (9)
**iRBD**	246.4±117.6 (16)	30.2±10.6 (5)	895.6±278.6 (5)	6849.0±3248.9 (4)	1.41±0.26 (4)
**PD**	207.9±144.9 (68)	46.5±31.8 (17)	656.9±206.2 (15)	-	-
**MSA**	319.4±199.5 (29)	50.6±24.5 (9)	792.7±363.8 (9)	-	-

The number of cases analyzed for each diagnostic group and each CSF biomarker are reported in brackets. All biomarker values, except the Aβ42/Aβ40 ratio are in pg/ml. For abbreviations see supplementary Table 1.

Supplementary Table 4. Percentage distribution of positive replicates across LBD groups*.

Diagnostic categories (positive/total samples)	Number of positive wells (samples, n) %		
**Incidental NP LB syn+ (6/7)**	2 (3) **50.0%**	3 (1) **16.7%**	4 (2) **33.3%**
**iRBD (18/18)**	2 (2) **11.1%**	3 (5) **27.8%**	4 (11) **61.1%**
**PAF (26/28)**	2 (1) **3.9%**	3 (3) **11.5%**	4 (22) **84.6%**
**DLB (47/48)**	2 (2) **4.2%**	3 (6) **12.8%**	4 (39) **83.0%**
**PD (67/71)**	2 (6) **9.0%**	3 (13) **19.4%**	4 (48) **71.6%**
**Total LB syn + (164/172)**	2 (14) **8.5%**	3 (28) **17.1%**	4 (122) **74.4%**

***Only the results of "diagnostic" positive runs are represented. For "undetermined" runs (1/4 positive wells see supplementary Table 6). For abbreviations see supplementary Table 2.

Supplementary Table 5. Phenoconversion from PAF to DLB, PD and MSA.

**Diagnosis at LP**	**Age at LP (yrs)**	**Time between first symptom(s) and phenotypic conversion (yrs)**	**Clinical diagnosis at last follow-up**	**α-syn RT-QuIC result**
PAF	48	7	MSA	Negative
PAF	69	7	DLB	Positive
PAF	52	9	PD	Positive
PAF, RBD	53	3	MSA	Negative
PAF	68	4	PD	Positive
PAF	68	3	PD	Positive

LP, lumbar puncture; for the other abbreviations see supplementary Table 1.

Supplementary Table 6. Percentage distribution of "unclear" results across diagnostic groups

	Unclear/Total runs (%)	Final result of repeated test	
		0/4 (%)	≥2/4 (%)
**Syn –**	**15*/ 193 (7.8)**	**14 (93.3)**	**1 (6.7)**
Syn- path controls	7* / 81 (8.6)	7	-
Clinical controls	5 / 62 (8.1)	4	1
PSP/CBS	3 / 31 (9.7)	3	-
**AD**	**5 / 60 (8.3)**	**3**	**2**
**MSA**	**5 / 33 (15.2)**	**5**	**-**
**Syn +**	**3* / 172 (1.7)**	**2 (66.7)**	**1 (33.3)**
Syn+ path controls	2* / 7 (28.6)	1	1
PD	1 / 71 (1.4)	1	-
**Total**	**28 / 439 (6.4)**	**24 (85.7)**	**4 (14.3)**

*One sample needed a further repetition because of a second unclear result.
For abbreviations see supplementary Table 1.

Supplementary Table 7. Performance of Syn RT-QuIC in positive samples repeated three times

		First result					
		2/4 (n=7*)		3/4 (n=4†)		4/4 (n=11§)	
**Diagnostic groups**		Second result (%)	Third result (%)	Second result (%)	Third result (%)	Second result (%)	Third result (%)
**syn +** (syn+ NP, DLB, PAF, RBD, PD)	0/4	-	-	-	-	-	-
	1/4	2 (28.6)	1 (14.3)	-	-	-	-
	2/4	2 (28.6)	3 (42.8)	-	-	-	1 (9.1)
	3/4	1 (14.2)	1 (14.3)	1 (20.0)	-	-	3 (27.3)
	4/4	2 (28.6)	2 (28.6)	3 (60.0)	4 (80.0)	11 (100)	7 (63.6)

*Includes 4 PD, 1 iRBD, 1 DLB and 1 syn+ NP; † Includes 2 PD, 1 iRBD, and 1 PAF; § Includes 4 PD, 2 iRBD, 4 DLB and 1 PAF. For abbreviations see supplementary Table 1.

Supplementary Table 8. Intra-batch and inter-batch coefficients of variation.

	# Batch	CV I max	CV AUC	CV lag phase
**Positive control**	1	19.82%	24.65%	14.98%
	2	7.43%	2.99%	21.79%
	3	8.12%	12.26%	16.85%
	4	11.50%	16.59%	22.97%
	5	8.88%	13.00%	18.48%
	6	18.15%	24.39%	32.25%
	7	22.17%	23.88%	25.88%
	8	10.04%	5.12%	12.25%
	9	16.21%	30.28%	32.06%
	mixed	15.81%	17.16%	20.40%
	**Inter-batch CV**	17.65%	21.16%	23.48%
**Negative control**	2	10.58%	4.21%	
	3	7.78%	8.66%	
	4	11.56%	10.99%	
	5	2.76%	1.32%	
	6	39.57%	28.72%	
	7	27.67%	21.92%	
	8	11.86%	11.28%	
	9	16.87%	6.92%	
	mixed	25.61%	22.83%	
	**Inter-batch CV**	36.41%	29.94%	

The intra-batch coefficient of variation (CV) of the maximum intensity of fluorescence (I max), area under the curve (AUC) and lag phase is expressed as percentage of the ratio between standard deviation and average.

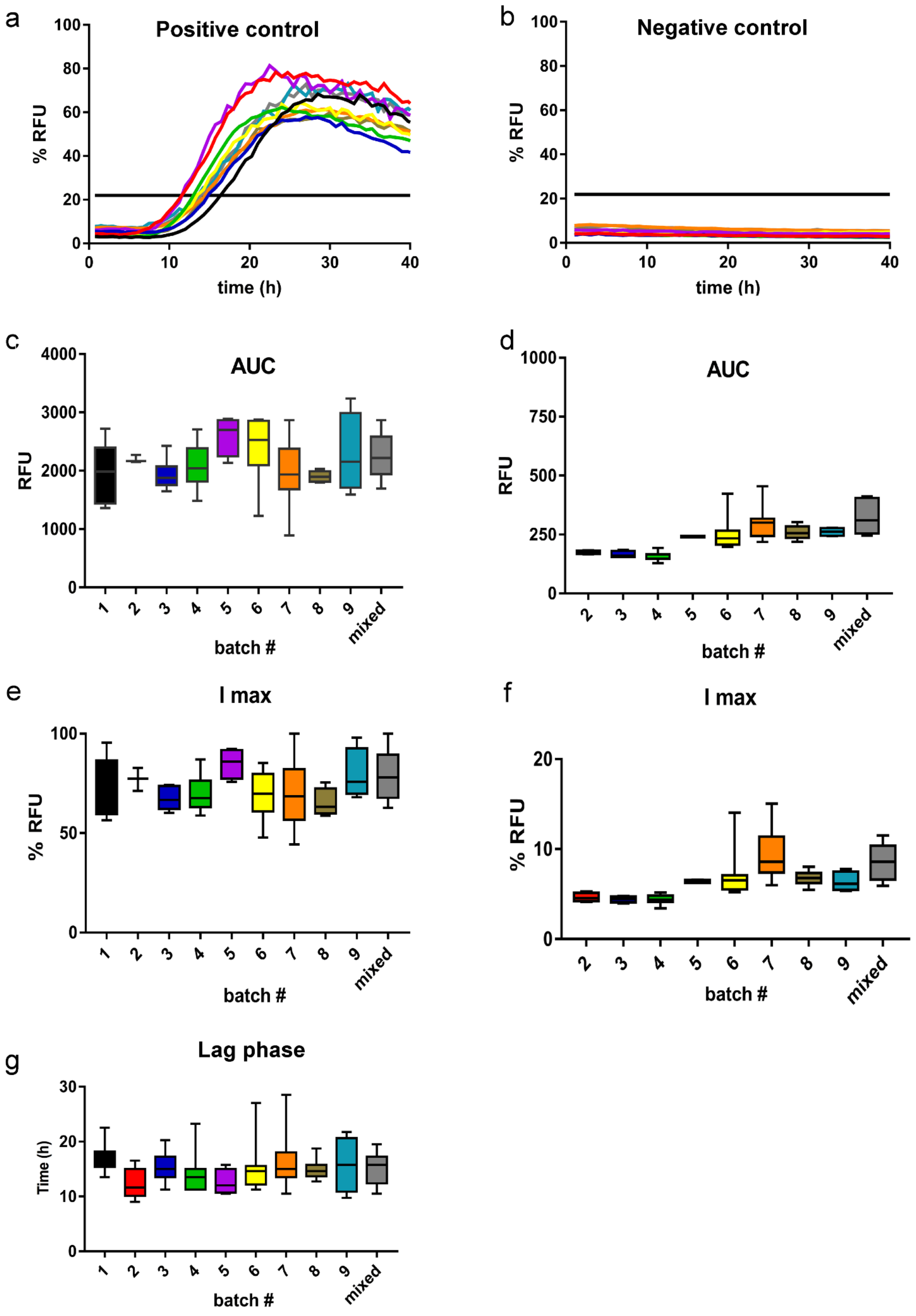

a
Positive control
% RFU
100
80
60
40
20
0
0
10
20
30
40
time (h)
b
Negative control
% RFU
100
80
60
40
20
0
0
10
20
30
40
time (h)
c
AUC
RFU
4000
3000
2000
1000
0
1
2
3
4
5
6
7
8
9
mixed
batch #
d
AUC
RFU
1000
750
500
250
0
2
3
4
5
6
7
8
9
mixed
batch #
e
I max
% RFU
100
50
0
1
2
3
4
5
6
7
8
9
mixed
batch #
f
I max
% RFU
20
10
0
2
3
4
5
6
7
8
9
mixed
batch #
g
Lag phase
Time (h)
30
20
10
0
1
2
3
4
5
6
7
8
9
mixed
batch #

Supplementary figure. Batch-to-batch and intra-batch variation of fluorescence signal induced by recombinant α-Syn aggregation in the RT-QuIC. Kinetic curves, AUC, and Lag phase of the same positive (**a, c, e**) and negative (**b, d, f**) control tested with different batches of recombinant α-Syn. Each colour depicts the performance of a different batch of α-Syn. Error bars in the kinetic curves were excluded in order to make the image easier to read. In the other graphs, error bars represent intra-batch variability, calculated for the positive control on, respectively 3 (batch #1), 2 (batch #2), 3 (batch #3), 4 (batch #4), 1 (batch #5), 2 (batch #6), 8 (batch #7), 2 (batch #8), 1 (batch #9), and 2 runs (mixed batches #2 + #6, and #8 + #9). For the negative control, error bars representing intra-batch variability, were calculated on, respectively, 1 (batch #1), 1 (batch #2), 3 (batch #3), 1 (batch #5), 2 (batch # 6), 8 (batch #7), 2 (batch #8), 1 (batch #9), and 2 runs (mixed batches #2 + #6 and #8 + #9). All control samples were run in quadruplicates. Differences in the dimension (n) of the runs per batch were related to the variability in the yield of recombinant α-Syn between batch preparations.
